# Comment on Kluckow M. Barriers to the Implementation of Newborn Pulse Oximetry Screening: A Different Perspective. *Int. J. Neonatal Screen.* 2018, *4*(1), 4

**DOI:** 10.3390/ijns4020013

**Published:** 2018-04-14

**Authors:** Thomas L. Gentles, Elza Cloete, Mats Mellander

**Affiliations:** 1 Green Lane Paediatric and Congenital Cardiac Service, Starship Children’s Hospital, 1023 Auckland, New Zealand; 2 Liggins Institute, University of Auckland, 1142 Auckland, New Zealand; 3 Queen Silvia Children’s Hospital, Department of Pediatric Cardiology, 41650 Gothenburg, Sweden

**Keywords:** pulse oximetry, screening, critical congenital heart disease, neonate

We read the review article by Kluckow M (Barriers to the Implementation of Newborn Pulse Oximetry Screening: A Different Perspective. *Int. J. Neonatal Screen.* 2018, *4*(1), 4) with interest and agree that this is an important subject to discuss. However, we do not agree with the view as to how pulse oximetry screening for critical congenital heart disease (CCHD) is best implemented and therefore would like to add to this discussion.

The article discusses two important issues in relation to pulse oximetry screening. The first question is whether the oximetry test is better packaged as an assessment of neonatal wellbeing rather than a way to detect CCHD. Kluckow argues for the first alternative as being preferable because he believes this way of doing it causes less anxiety for parents. The second question Kluckow discusses is whether pulse oximetry testing is best delivered as a nationwide screening program or on an individual hospital basis. Kluckow is of the opinion that the second alternative is more likely to be successful (at least in Australia).

If it were not that the first question was closely linked to the second and more important one, the answer would be fairly straightforward, as pulse oximetry testing may detect babies with systemic illness including respiratory illness and infection at a greater rate than it does CCHD [1]. The second question, whether pulse oximetry testing is best delivered as a universal screening program or on an individual basis, is more complex. The answer in part depends on local factors. Who is responsible for the newborn assessment and how would oximetry testing be incorporated into the newborn examination? Who will pay for the time, equipment and disposables? In New Zealand, where the newborn assessment is undertaken by midwives with a number of competing priorities, the incorporation of an oximetry test into the newborn examination is unlikely to be universal. Certainly in a pilot study involving several different hospital care settings, the uptake of oximetry testing ranged between 45% and 85% despite an intensive targeted staff education campaign (preliminary data).

Universal pulse oximetry has the potential to improve health outcomes in the most disadvantaged newborns: those whose mothers are less likely to obtain quality obstetric care and who may not obtain any obstetric care until late in pregnancy [2]. These factors, in addition to maternal obesity, which is also linked to social deprivation, mean that babies of disadvantaged mothers are less likely to have an antenatal diagnosis of CCHD. In New Zealand as elsewhere, a significant number of newborns die or have lasting damage because of the late diagnosis of CCHD [3]. Both New Zealand and Australia have minority indigenous and immigrant populations whose health outcomes fall far below those of the dominant culture. Whichever way the pulse oximetry test is packaged, it is important that its reach and impact are understood and iteratively improved upon. Without an ongoing audit of uptake and knowledge of the population-based (rather than hospital-based) rate of late or undiagnosed CCHD, it is not possible to assess whether a program is effective. An ad hoc roll out of oximetry testing in large tertiary hospitals is likely to target the children of higher income families who are the most likely to have an antenatal diagnosis. Moreover, those born in peripheral hospitals are, in our experience, more likely to receive a delayed diagnosis of CCHD and are therefore most likely to benefit from screening. These same factors are very likely to have contributed to the marked difference in CCHD detection rates in US centers where mandatory screening programs were associated with a 33% reduction in infant death from CCHD compared to no reduction in those states who adopted non-mandatory policies [4]. It is important that these issues are addressed by the broader community to ensure that oximetry testing is delivered in a way that reduces inequity rather than magnifies it.

Neonatal pulse oximetry screening for CCHD has the greatest potential to be effective if implementation is universal. If it is left to each hospital to implement screening, there is a risk that parts of the population who are most likely to benefit from postnatal screening for CCHD will be least likely to receive it.
